# Challenges Associated with Monitoring Long-Term Health Effects After a Major Accident: Lessons from a Large Chemical Fire in England

**DOI:** 10.3390/ijerph23070894

**Published:** 2026-07-10

**Authors:** Brandon Parkes, Katie Hopgood, Siobhan Farmer, Bethan Davies, Frédéric B. Piel

**Affiliations:** 1 Small Area Health Statistics Unit (SAHSU), Department of Epidemiology & Biostatistics, School of Public Health, Imperial College London, 90 Wood Lane, London W12 0BZ, UKbethan.davies06@imperial.ac.uk (B.D.); 2 MRC Centre for Environment & Health, Department of Epidemiology & Biostatistics, School of Public Health, Imperial College London, 90 Wood Lane, London W12 0BZ, UK; 3 NIHR Health Protection Research Unit in Environmental Exposures & Health, School of Public Health, Imperial College London, 90 Wood Lane, London W12 0BZ, UK; 4 Gloucestershire County Council, Shire Hall, Westgate Street, Gloucester GL1 2TG, UK; katie.hopgood@gloucestershire.gov.uk (K.H.); siobhan.farmer@gloucestershire.gov.uk (S.F.)

**Keywords:** accidental fire, community engagement, ecological epidemiological study

## Abstract

**Highlights:**

**Public health relevance—How does this work relate to a public health issue?**

- Large accidental fires can lead to exposure to chemicals and potential health risks.
- Beyond short-term emergency responses, long-term health risks also need to be considered.

**Public health significance—Why is this work of significance to public health?**

- This work presents a real-world example of a 20-year follow-up following a large chemical fire.
- Rigorous scientific evidence from a retrospective epidemiological study may not alleviate concerns of members of the local community.

**Public health implications—What are the key implications or messages for practitioners, policy makers and/or researchers in public health?**

- Careful and realistic plans for monitoring long-term health impacts need to be considered early after such an incident.
- Clear communication with all key stakeholders throughout the study period and engagement with local residents are essential to maintain trust in study findings.

**Abstract:**

Major incidents, such as large fires and floods, often trigger a complex series of environmental and health risk assessments involving multiple institutions and authorities. They can also lead to long-lasting concerns from members of the local communities about the possible impact on their health and environment. Here, we use the example of a large fire that occurred in 2000 at a chemical storage site in Sandhurst, Gloucestershire, England, to contrast evidence from official reports and scientific studies with the expectations of members of the local community. We reflect on how the handling of such an incident has evolved over the last two decades and how public involvement can be further improved in the future. Firstly, we present the results of a 20-year follow-up (2001–2020) retrospective small-area ecological epidemiological study with rates of overall cancer incidence, all-cause mortality and hospital admissions for respiratory disease in the exposed area compared with rates in the South West region of England. We also studied the ten years preceding the fire (1991–2000). Secondly, we discuss the limitations of these findings to alleviate the concerns of the local community. Finally, we use this case study to make recommendations about how to better manage this balance between scientific evidence and public concerns for future incidents. In line with earlier reports, the 20-year follow-up study did not identify any major increase in relative risks for cancer or mortality. Although an increase in admissions for respiratory disease was identified, this was also observed before the fire, suggesting that this may be due to other local factors. Despite these findings, members of the local community still believe that some local cases of cancer were caused by the fire. As rigorous as a long-term epidemiological study can be, it cannot determine causality between an incident and individual cases for a given health outcome, nor can it make up for the absence of data not—or only partly—collected after the incident (e.g., individual-level health register). This case study further highlights that engaging with local communities and managing their expectations and planning potential long-term follow-up studies immediately after a major incident should be carefully considered.

## 1. Introduction

With the global production and storage of chemicals rapidly growing, major chemical fires happen regularly worldwide [1]. Examples of large fires involving potential health risks for the local population due to airborne exposures over the last few decades include the 1986 Sandoz warehouse fire in Switzerland [2]; the 2015 Tianjin explosions in China [3]; the 2019 Lubrizol chemical plant fire in Rouen, France [4]; the 2020 Beirut explosion in Lebanon [5]; and the 2024 Biolab fire in Georgia, USA [6]. Despite strict industrial safety regulations, it is essential to learn from such incidents to be better prepared in both emergency responses and long-term environmental and health monitoring.

A wide range of methods, including individual-level monitoring and ecological studies (e.g., small-area studies) [7], can be used prospectively or retrospectively to study long-term health effects from various environmental exposures following such incidents. Immediate responses to such events need to be rapid, appropriate and proportional and often focus on short-term environmental and health risk assessments. Nevertheless, long-term risks also need to be considered, and data collection required to support potentially decades-long investigations needs to be carefully planned. In parallel, because of their nature and scale, such major events often lead to substantial concerns within the local communities affected. Here, we use a 20-year follow-up ecological study following a large chemical fire in a waste storage facility in Sandhurst, Gloucestershire, England, as an example of the challenges of retrospectively addressing the concerns of the local communities with population-level epidemiological data. Our aim is to use this case study to reflect on strategies that can be adopted to improve planning of long-term investigations after a major chemical fire incident. Details of the incident, health monitoring activities undertaken and previous reports are summarised in the Supplementary Material (Background S1). A timeline of the incident, main reports and public engagement activities is presented in the Supplement (Figure S1). In brief, a large fire, involving a complex mixture of over 177 tonnes of chemicals, broke at a storage site located in Sandhurst, Gloucestershire, in October 2000. Various health and environmental monitoring activities were undertaken. Although earlier reports did not identify major health or environmental risks, the local public health team committed to continue health monitoring over a period of twenty years to provide reassurance to concerned members of the local community. In line with this original commitment, the local authority (Gloucestershire County Council, GCC) commissioned in 2019 a follow-up study from the Small Area Health Statistics Unit (SAHSU) covering the period 2001–2020. The purpose of this long-term independent study was to assess health risks for mortality, cancer and respiratory disease, using population-level data in order to address ongoing concerns of the community.

## 2. Materials and Methods

No individual-level exposure or health data were available for this study. As such, the small-area ecological study design, already chosen in the original 2011 SAHSU study [8], was considered to be the most suitable methodology for this health risk assessment. A comparison of the methodology used in the 2011 and 2023 SAHSU studies is summarised in Table S2. Members of the community were not involved in the study design. SAHSU conducted the study independently but worked with GCC to engage with the local community to share the findings and to reflect on their public health implications.

**Figure 1:**
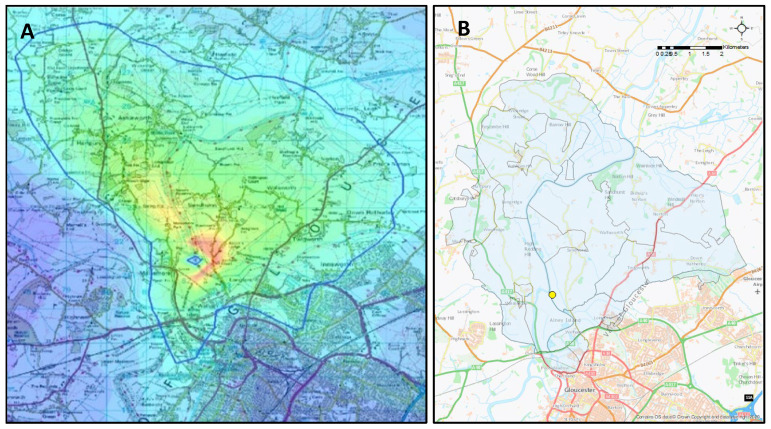
(**A**) Image of the modelled plume of the fire, reproduced with permission from Ward and Stone (2006) [9]. (**B**) Area (blue shade) deemed to be potentially exposed to the plume of the fire at ground level. The yellow dot indicates the site of the fire.

Ground-level modelling of the plume from the fire was originally performed by researchers at Birmingham University [9]. Given data retention policies and storage limitation under the General Data Protection Regulation (GDPR) in place in the UK, no copy of the report providing details of the modelling methodology could be accessed. The modelled plume spread predominantly to the North East, but stormy conditions following the start of the fire suggested that all areas around the storage site were likely to have been exposed at some point during the sixteen hours of burn (Figure 1A). As a result, we defined the exposed population as those living in the administrative areas immediately surrounding the site of the fire, extending approximately five kilometres to the North and East, as used in the 2011 study (Figure 1B).

We retrospectively performed a small-area ecological epidemiological study using population and health data recorded by Census Output Areas (COAs), which are the smallest geographical units at which all census data are recorded (the average population of a COA in the 2011 Census was 309) [10]. The modelled plume shown in Figure 1 overlapped with nineteen COAs, which were used to define the exposed population and our study area. In line with standard practice, our reference area was defined as the South West region of England [11] excluding the study area (17,625 COAs in 2011) (Supplementary Figure S2 and Table S3).

The study period covered the twenty years after the fire (from January 2001 to December 2020). We also performed the analysis for ten years prior (1991–2000) to the fire as a reference period.

Age- and sex-specific COA-level population data were obtained from the Office for National Statistics (ONS) annual mid-year population estimates [12]. We extracted annual COA-level count data, by sex and age for three health outcomes: (i) incident cancer diagnoses (all-cause) from the National Cancer Registration Statistics held by NHS England; (ii) all-cause mortality from ONS Mortality Registrations; and (iii) in-patient hospital admissions for respiratory disease (primary diagnosis code using International Statistical Classification of Diseases and Related Health Problems 10th Revision (ICD-10) [13] classification beginning with “J”) from NHS England Hospital Episode Statistics (HES). HES cover admissions at all NHS hospitals in England [14]. Due to low numbers of cases per year, data for each health outcome were aggregated into six five-year periods: 1991–1995, 1996–2000, 2001–2005, 2006–2010, 2011–2015 and 2016–2020. At the time of the study, cancer registrations were not available beyond 2017; consequently, the cancer data were analysed in the following periods: 1991–1995, 1996–2000, 2001–2005, 2006–2010, 2011–2015 and 2013–2017 with a three-year overlap in the two most recent periods. Specific cancers and causes of death were not investigated because of concerns about low numbers potentially giving spurious results. Nevertheless, as in the 2011 report, a sensitivity analysis considering lung, breast, prostate, colorectal cancer and leukaemia combined was also conducted. Ages were limited to 0–84 years for cancer registration and hospital admission episodes as data have been shown to become less reliable for the population aged over 85 years [15,16,17,18].

Adjustments for differences in demographics (age and sex structure of the population) were made using indirect standardisation [19,20,21] based on age–sex categories with age in five-year bands. Since deprivation is associated with a number of adverse health outcomes [22], it is important to adjust for it as a potential confounder. The Carstairs index is a measure of socio-economic status derived from census data, originally defined using data from the 1981 Census, and available at COA level for all subsequent censuses [23,24]. We used the 2001 and 2011 Carstairs indices divided into quintiles as covariate data representing deprivation. Adjusting for smoking prevalence throughout the study period was not possible due to the absence of a long-term consistent measure of smoking in England at the COA level. One of the available proxies for smoking at COA level is CACI data on tobacco expenditure [25,26] from 2011 onwards (© Copyright 1996–2014 CACI Limited, London, UK). We could therefore only apply adjustment for smoking using the CACI tobacco expenditure data for the years 2011–2020.

We compared rates of cancer incidence, mortality, and hospital admissions with respiratory disease in the exposed areas to those in the reference area to produce indirectly standardised mortality and morbidity relative risks (RRs) and 95% confidence intervals (CIs) for the study area. Analyses were performed separately for males and females and both sexes combined. All analyses were conducted in R 3.2.1 (R Foundation for Statistical Computing, Vienna, Austria) [27].

This long-term study stemmed from concerns expressed by the local community. Two years after the fire, local residents reported various health problems. Later on, concerns were raised about cancer risks based on individual cases of thyroid and oesophageal cancers. Throughout the conduct of this study, GCC compiled messages related to health concerns following the fire. Representatives from SAHSU and GCC presented and discussed the study findings with members of the community during an in-person event held in early 2023. The outputs from these discussions and follow-ups were used to reflect on challenges associated with such a long-term study and to inform responses to future incidents.

## 3. Results

The results of this long-term small-area ecological study were overall consistent with the findings of the 2011 study for the three health outcomes considered [8].

### 3.1. Cancer Incidence

**Figure 2:**
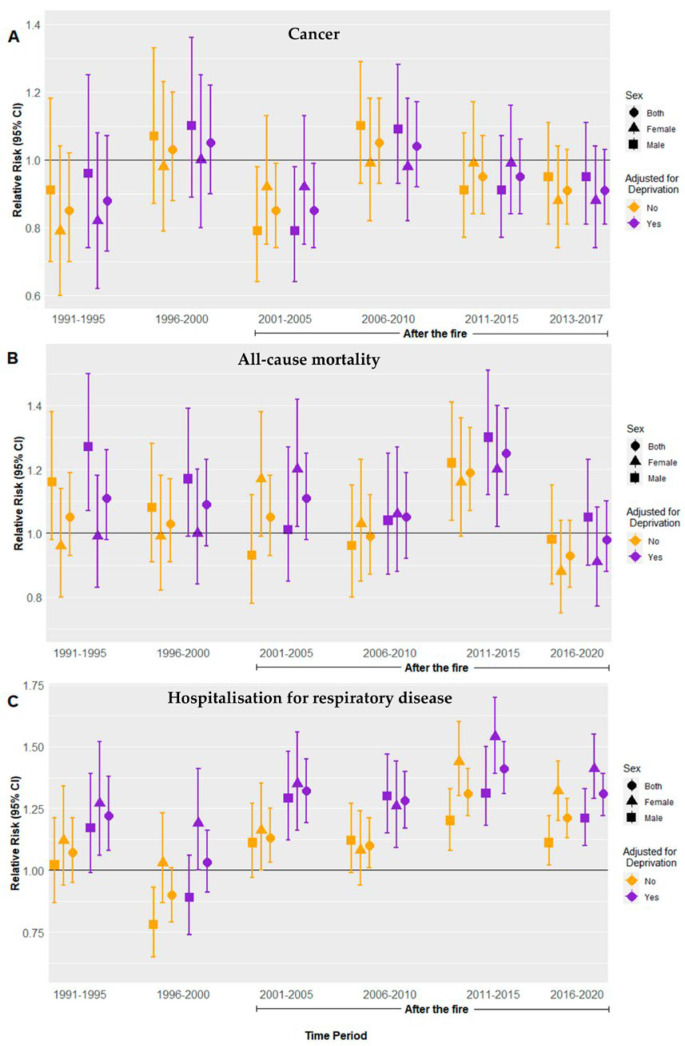
Indirectly standardised relative risk for: (**A**) all cancer registrations; (**B**) all-cause mortality; and (**C**) hospitalisation for respiratory disease in the study area 1991–2020. Excluding 85+ age categories for (**A**,**C**). Reference period 1991–2000 (prior to the fire). For tables of relative risks, see Supplementary Tables S1–S18.

The relative risks for all cancers between 1991 and 2017 are presented in Figure 2A. No statistically significant increase in cancer registrations was seen for any of the time periods considered, with or without adjustment for deprivation and smoking (Tables S4–S9). In 2001–2005, the observed numbers of cancer registrations in males and in both sexes were statistically significantly lower than the expected counts (Table S6). For the 2006–2010 period, there was a non-statistically significant increase in the unadjusted (9.8%; 95%CI: −6.7–29.2%) and adjusted (9.1%; 95%CI: −7.3–28.4%) RR of all cancer registrations for males (Table S7). A similar pattern was observed before the fire in the period 1996–2000 (Table S5). The results were consistent when considering a subset of lung, breast, prostate, colorectal cancer and leukaemia combined in our sensitivity analysis (results not shown).

### 3.2. All-Cause Mortality

There were statistically significant excess risks for mortality in males (33%; 95%CI: 15–55%), females (21%; 95%CI: 3–42%) and both sexes combined (27%; 95%CI: 14–42%) in 2011–2015 (Table S14) after adjustment for deprivation and smoking (Table S12). Males in 1991–1995 (Table S10) and females in 2001–2005 (Table S12) also had a statistically significant excess risk of all-cause mortality when adjusted for deprivation (Figure 2B). Excesses of mortality (11% and 9% respectively) for both sexes combined for 1991–1995 (Table S10) and 1996–2000 (Table S11) were not statistically significant. These excesses were similar to those seen in 2001–2005 (11%) (Table S12) and 2006–2010 (5%) (Table S13). Adjustments had a substantial impact on the findings, particularly for males, with some relative risks shifting from <1 to >1.

### 3.3. Hospitalisation for Respiratory Disease

Unadjusted findings were statistically significant for males in 1996–2000 (RR < 1); for males and females from 2011–2015 onwards (RR > 1); and for both sexes from 2001–2005 onwards (RR > 1) (Figure 2C, Tables S16–S21). All adjusted excesses were statistically significant for males, females and both in all the time periods studied, apart from males in 1991–1995 and males and both sexes in 1996–2000. The largest excess risk for both sexes combined was 47% (95%CI: 36–58%) in 2011–2015 after adjusting for deprivation and smoking (Table S20).

### 3.4. Community Engagement

The reaction of the community representatives to the presentation of these findings was mixed. Some were interested in the results of this long-term follow-up to which GCC had committed. Others expressed concerns that authorities had originally intimated that a different, individual-level follow-up study would take place (i.e., tagging of GP records, which would potentially have allowed for longer-term cohort or case–control study design). This study design was proposed but was not progressed due to the authorities being unable to obtain a sufficient sample size from the local community due to opt outs when seeking consent. It appears that in making the decision to not proceed with the original study design, and instead use the small-area study methodology chosen to monitor the health of the population, members of the community had not been made aware or had not fully understood the reasons for this or the limitations of the chosen study design. Once the uncertainties of the study results were outlined, there was some anger and disappointment that it did not explain whether their personal experiences were linked to the incident or not, leading to suggestions that the report was not fit for purpose and that the lived experience of residents diverged from the evidence presented. Attendees openly shared the psychological impact of both the incident itself, the commitments that they believe were made and not followed through, and the hope of answers through a long-term study but needing to live with revisiting trauma as a result. The discussion also led to questions about the impact of population movement in and out of the area, and the possibility of conducting a further study covering a longer period than the twenty years considered here.

## 4. Discussion

The underlying assumptions behind the commitment to conduct a long-term study were that (i) over time, an epidemiological study was expected to benefit from larger numbers of cases and larger numbers of controls, compared to a reference area, therefore leading to a higher statistical power to detect differences in health risks; and (ii) the findings would provide scientific evidence to reassure the local community. Although this long-term study, covering a period of 20 years after the incident, found limited evidence of obvious health risks related to the incident, it raises important points about the limitations of retrospective studies and their scope to address the concerns of local residents affected [28].

In summary, we did not find a statistically significant increase in the incidence of cancer following the incident, and we found some excess risk for both sexes following the incident in relation to all-cause mortality and hospital admissions for respiratory diseases, but similar statistically significant excess risks were also present before the fire. These findings are consistent with the initial joint investigation conducted by the Environment Agency and Health & Safety Executive in 2001 [29], and with the earlier 2011 SAHSU study [8]. A possible explanation for the excess risk of all-cause mortality, and potentially respiratory hospital admissions, is the presence in the study area of a care home specialising in dementia, which opened in the 1990s and closed in 2018 [30,31,32].

Although our focus here was on chemical fires, there is a wider range of similar incidents that can lead to airborne exposure of the local population. The health effects of exposure to smoke from forest fires have been estimated more extensively [33,34] including country-wide studies of cancer incidence in people exposed to wildfire smoke in Canada [35] and Brazil [36]. We should therefore be better prepared to respond to such incidents and to monitor environmental and health impacts. Regulation improvements in tracing of chemicals and compliance checks, together with refined air quality monitoring, should help to better identify the toxicity risks if a similar event occurred now [37,38]. Furthermore, the Air Quality in Major Incident service (AQinMI), set up in 2009, provides more accurate exposure data in the UK [39].

It is essential to establish from the onset the best study design and data required to monitor short- and long-term environmental and health risks, as appropriate. In the absence of individual-level data for the exposed population, it will not be possible to precisely account for the variability in the exposure of the local residents during and after the incident (e.g., hours inside/outside, workplace exposure), nor for population movement in and out of the study area to be taken into account. Regression-based methods could be considered as they offer more flexibility in adjusting for confounders.

Rapidly establishing a health register of residents potentially exposed to pollution from the incident can enable close monitoring of exposed individuals, through active (e.g., surveys and exposure assessments) or passive (e.g., administrative health records) approaches, independent of migration or demographic changes, which might dilute health effects [40]. In England, this approach can be led by local authorities in collaboration with the UK Health Security Agency, but establishing a register is time-consuming, costly and challenging. This was attempted in the Sandhurst case, but was not fully implemented, partly due to lack of engagement from members of the local community [41]. In the absence of a health registry of people exposed to the Sandhurst fire, no individual-level data were available to assign detailed exposure accounting, for example, for hours of exposure indoors and outdoors, workplace exposure or movement during and after the fire.

Population movement means that the residents in the affected area change over time. The twenty-year study period would theoretically allow identification of long-term health effects associated with the incident. Nevertheless, the fact that the local population changed over time made this challenging to assess. In this case, the population change was high, with a 39.2% increase between 2000 and 2020 compared to 14.2% for the whole of England [42]. We had no way to monitor inward or outward migration in this analysis that used place of residence at the time of the health outcome (cancer diagnosis, death, hospital admission) to define the study population. This limitation was discussed with the community who understood that this had ‘diluted’ the study population and were disappointed that it made it harder for the true effects to be measured.

Regular measurements of specific pollutants in soil and water throughout the study period would have been required to conduct a comprehensive long-term environmental and health risk assessment. By limiting our definition of exposure to the population whose place of residence was covered by the modelled plume, we have only considered exposure to airborne pollution from the fire. Residents had registered concerns about exposure to toxic chemicals from the site in the years after the site was opened in 1977 but before the fire [8]. Although assessing chronic exposure would require a different study design, it would also require long-term measurements of pollutants and accurate health data. Citizen science approaches can now be used to involve members of the local community in providing samples [43,44]. Nevertheless, significant costs can be associated with the storage, processing and analysis of samples collected.

Over twenty years after the CSG incident, local residents still have concerns and their own hypotheses about health outcomes that they attribute to the incident. While scientific studies like this provide valuable evidence at the population level, they may not alleviate the concerns of the local community. As is the case in cluster investigation studies [45], excellent communication is required at all stages of the process. Risk communication is essential for the adequate management of a major incident such as the Sandhurst fire [46,47]. Public attitudes towards risk and towards government are evolving, while the pace and reach of communication (e.g., social media) has considerably increased. As part of the UK Government Resilience Framework, the Cabinet Office has developed a practical toolkit, based on a seven-step risk communication strategy [48], while the US Centers for Disease Prevention & Control (CDC) have a Crisis & Emergency Risk Communication (CERC) manual, covering topics such as the psychology of a crisis and working with the media [49]. The overall short-term goal of community engagement in an emergency is to provide the most people with the information and options that they need to make decisions and take actions that save lives and lead to recovery. Trying to convince people or groups who are sceptical or adversaries would be a waste of time and resources in an emergency [50]. Nevertheless, beyond the emergency response, understanding the concerns of these members of the local community, including through psychosocial and mental health care, may help in refining the communication strategy and consolidating a relation of trust [51]. It is critical to listen to the concerns of local community members early on; to engage with local representatives throughout the duration of any investigation; and to develop trust with the community. A qualitative study collating the experiences and feelings of those who experienced the incident first hand would help inform designs of epidemiological studies following comparable events in the future. Although public and community involvement and engagement practices may have improved recently, this is not consistently reflected in guidance documents and in post-incident monitoring [52].

Whether retrospective or register-based, careful consideration needs to be given to data collection, retention and processing by relevant authorities, including data protection and continuity across organisational changes, as well as sustainable funding sources and communication with potential participants about study design, duration, limitations and possible impacts of prolonged involvement. Such long-term investigations are complex and usually involve multiple stakeholders. Given the timespan between the incident and the initiation of such a long-term follow-up study, our work highlighted the importance of good archiving by stakeholders to keep internal knowledge of the incident and justifications of the decisions made. Whether decisions are taken in collaboration with community members or not, this is essential to be accountable. At the community event, the authors were able to assure those present that emergency response guidance is now clearer on record keeping and logging decisions. For example, electronic records tend to be easier to track and search than paper records, and public inquiries into major incidents have stressed the importance for incident responders to keep clear logs of decisions and to ensure that dynamic risk assessments and actions taken are recorded [53]. These principles should also be applied to decisions around research and evaluation studies that examine the impacts upon individuals and communities over an extended period. This needs to occur at both the initial response phase (e.g., setting up of a register of individuals affected) and into the recovery phase of an incident (agreeing what studies will or will not be carried out to understand health impacts).

## 5. Conclusions

This case study highlights the complexities of conducting long-term health monitoring after a major chemical incident. It also emphasised the possible mismatch between the expectations of the local community and the scientific evidence that cannot be provided, particularly in relation to causality for individual cases. Careful preparation, clear guidance and close cooperation between investigators and residents immediately after an incident are essential to improve the likelihood of identifying and collecting samples and individual-level data on likely exposure and health impacts. Investigators can then work with community representatives and other stakeholders to agree on a study design that is both feasible and designed to address the concerns of the exposed population, and to communicate transparently about decisions taken. Further guidance about best practices to achieve this would be valuable.

## Data Availability

Population data available from [10,12]; cancer incidence data from National Cancer Registration Statistics held by NHS England; mortality data from ONS Mortality Registrations; Hospital Episode Statistics (HES) available on request from NHS Digital [14].

## Supplementary Materials

The following supporting information can be downloaded at: https://www.mdpi.com/article/10.3390/ijerph23070894/s1, Background S1. Overview of the Sandhurst incident, health monitoring activities undertaken and previous reports; Methods S1. Changes in Census Output Areas (COAs) between the 2001 and 2011 Censuses; Figure S1. Timeline of the incident and main reports and public engagement activities; Figure S2. Maps of the study and comparison areas; Table S1. Toxicity of the chemical substances involved in the fire at the CGS storage site; Table S2. Comparison of the methodology used for the 2011 and 2023 Small Area Health Statistics Unit (SAHSU) studies; Table S3. Comparison of the study area with the South West of England; Table S4. All cancers 1991–1995; Table S5. All cancers 1996–2000; Table S6. All cancers 2001–2005; Table S7. All cancers 2006–2010; Table S8. All cancers 2011–2015; Table S9. All cancers 2013–2017; Table S10. All-cause mortality 1991–1995; Table S11. All-cause mortality 1996–2000; Table S12. All-cause mortality 2001–2005; Table S13. All-cause mortality 2006–2010; Table S14. All-cause mortality 2011–2015; Table S15. All-cause mortality 2016–2020; Table S16. Respiratory hospital episodes 1991–1995; Table S17. Respiratory hospital episodes 1996–2000; Table S18. Respiratory hospital episodes 2001–2005; Table S19. Respiratory hospital episodes 2006–2010; Table S20. Respiratory hospital episodes 2011–2015; Table S21. Respiratory hospital episodes 2016–2020 [54,55].

## Abbreviations

The following abbreviations are used in this manuscript:

AQinMI	Air Quality in Major Incident service
CSG	Cleansing Service Group
CDC	Centers for Disease Control and Prevention (US)
CERC	Crisis & Emergency Risk Communication
COA	Census Output Area
GCC	Gloucester County Council
GDPR	General Data Protection Regulation (UK)
GP	General Practitioner
HES	Hospital Episode Statistics
NHS	National Health Service (UK)
SAHSU	Small Area Health Statistics Unit
ONS	Office for National Statistics
