# Supplementary material for: Challenges Associated with Monitoring Long-Term Health Effects After a Major Accident: Lessons from a Large Chemical Fire in England
Source: Int J Environ Res Public Health. 2026 Jul 10;23(7):894. doi: 10.3390/ijerph23070894 (PMC13409744; doi:10.3390/ijerph23070894)
Supplement: Supplementary file 1 [file ijerph-23-00894-s001.zip › ijerph-4219835-supplementary.pdf]

Supplementary Material

Challenges associated with monitoring long-term health effects after a major accident: lessons from a large chemical fire in England

Brandon Parkes, Katie Hopgood, Siobhan Farmer, Bethan Davies & Frédéric B. Piel

Contents

Supplementary Material 1

Supplementary Background 3

Background S1. Overview of the fire at the chemical storage site in Sandhurst, Gloucestershire, health monitoring activities undertaken and previous reports 3

Supplementary Methods 5

Methods S1. Changes in Census Output Areas (COAs) between the 2001 and 2011 Censuses 5

Supplementary Figures 6

Figure S1. Timeline of the incident and main reports and public engagement activities 6

Figure S2. Maps of the study and comparison areas. 7

Supplementary tables 8

Table S1. Toxicity of the chemical substances involved in the fire at the CGS storage site 8

Reference 10

Table S2. Comparison of the methodology used for the 2011 and 2023 Small Area Health Statistics Unit (SAHSU) studies. 11

Table S3. Comparison of the study area with the South West of England 12

Table S4. All cancers 1991-1995 13

Table S5. All cancers 1996-2000. 13

Table S6. All cancers 2001-2005. 13

Table S7. All cancers 2006-2010. 13

Table S8. All cancers 2011-2015. 14

Table S9. All cancers 2013-2017. 14

Table S10. All-cause mortality 1991-1995. 14

Table S11. All-cause mortality 1996-2000. 15

Table S12. All-cause mortality 2001-2005. 15

Table S13. All-cause mortality 2006-2010. 15

Table S14. All-cause mortality 2011-2015. 16

Table S15. All-cause mortality 2016-2020. 16

Table S16. Respiratory hospital episodes 1991-1995 16

Table S17. Respiratory hospital episodes 1996-2000 17

Table S18. Respiratory hospital episodes 2001-2005 17

Table S19. Respiratory hospital episodes 2006-2010 17

Table S20. Respiratory hospital episodes 2011-2015 18

Table S21. Respiratory hospital episodes 2016-2020 18

Supplementary Background

Background S1. Overview of the fire at the chemical storage site in Sandhurst, Gloucestershire, health monitoring activities undertaken and previous reports

The Sandhurst, Gloucestershire incident occurred in October 2000, when a large fire broke at a waste storage site owned by Cleansing Service Group (CSG) Ltd. The weather was stormy with heavy rain and wind speeds up to 60 mph [1]. On 3 November, the River Severn flooded the CSG site complicating the clean-up. The cause of the fire has never been established and although inventories of the substances involved in the fire are available [1,2], it is hard to know with accuracy the quantities that were combusted, dispersed by the firefighting activities, or washed away by the subsequent flood. Investigators estimated that over 177 tonnes of organic solvents were combusted including 1.1 tonnes of substances defined as very toxic (Table S1) [1]. Other chemicals such as chlorinated solvents, pesticides, waste laboratory chemicals, mercury, zinc and cyanide, were also involved in the incident. The main toxic risk was thought to be the release of approximately 30 tonnes of hydrogen chloride (HCl) gas created from the combustion of various chlorinated solvents (e.g. trichloroethylene and methylene chloride) [1]. Given the mixture of chemicals involved and the uncertainty about the quantities and routes of exposure, it is almost impossible to define a clear toxicological profile of this incident.

Concerns from the local community stemmed from a history of complaints regarding odours and nuisance at the waste treatment facility preceding the incident, and the incident itself. Approximately 60 local residents were evacuated during the fire and were allowed to return home later that day [1]. Following the fire, there were numerous reports of illness from local residents. HCl can cause irritation to the skin and eyes, headaches and wheeze [3]. Short-term health effects of other chemicals combusted include headaches, nausea, nose and throat irritation and breathing difficulty [2], some of which were symptoms reported by residents and emergency service personnel immediately following the fire. Nobody required admission to hospital suggesting the acute effects of exposure did not cause death or serious acute morbidity [4].

Various subsequent health monitoring activities were undertaken. These included: i) sharing the chemical inventories with local GP surgeries and residents, and monitoring the symptoms reported by residents; ii) sending health questionnaires to local residents and residents of two comparison areas; iii) testing blood and urine of site employees and other staff who had visited the site; iv) testing of a total of 500 air, water and soil samples which showed no significant levels of contamination; v) modelling the dispersion of the plume to help identify potentially exposed populations [2]; and vi) setting up a biological monitoring programme in early 2001 to analyse hair samples from 194 local residents [2]. The analysis of the samples showed that some residents had raised levels of heavy metals (chromium, manganese, nickel and selenium) but there was no association between the participants with raised trace element levels and those who reported health problems after the fire or their place of residence [2]. A 2001 Health & Safety Executive (HSE) report conducted concluded that "the plume was unlikely to have contained toxic material sufficient to give an HSE dangerous dose off-site."[1]

Nevertheless, suggestions about potential carcinogenic and mutagenic properties of some of the combusted chemicals raised concerns that there may be long-term detrimental health effects in the population exposed to the plume from the fire not highlighted by the environmental surveys or biomonitoring that would only become apparent in the decades following the fire. Consequently, the local public health team (Gloucestershire Primary Care Trust, transferred to Gloucestershire County Council (GCC) in 2012) committed to monitoring the community's health for a period of twenty years. In 2010, they commissioned the Small Area Health Statistics Unit (SAHSU) at Imperial College London to conduct an investigation into potential health risks associated with this incident with the primary aim to retrospectively assess if the exposed population had a greater incidence of cancers and deaths than expected [5]. Given the wide range of substances combusted in the fire, the uncertainty

about exact quantities stored/combusted, and the relatively small population exposed, relative risks for all health effects associated with each substance could not feasibly be calculated. Consequently, registrations of all types of cancer and overall mortality rates were considered as suitable outcomes to meet the primary aim of the study. In addition, given the focus on airborne exposure from the plume, counts of hospital admissions for respiratory disease were also considered. The report produced by SAHSU in 2011 [5] therefore provided indirectly standardised relative risks for cancer registrations (1974-2005), all-cause mortality (1989-2006) and hospital admissions for respiratory disease (2001-2005) for the area exposed to the modelled plume from the fire compared to the South-West region of England (reference population). The study found no clear evidence of excess in cancer registrations or mortality following the fire but rates of respiratory hospital admissions for 2001 to 2005 were approximately 33% higher than expected. Despite these official findings being reassuring overall, some local residents remained sceptical as they attributed specific cases of cancer (e.g. thyroid or oesophagus) to the incident.

References

1. Heath and Safety Executive, *Report for the Deputy Prime Minister, the Right Hon John Prescott MP, into the major fire on 30 October 2000 at Cleansing Service Group Ltd Sandhurst*. 2001.
2. Ward, N. and C. Stone, *Trace Elements in Human Hair as an Indication of the Exposure of Individuals of the Sandhurst Area of Gloucestershire Following a Chemical Fire*. 2006.
3. ACUte Exposure GUideline Levels, *Hydrogen Chloride'.* Acute Exposure Guideline Levels for Selected Airborne Chemicals: Volume 4, 2004. **4**: p. 77.
4. Whitfield, A. (2002), *COMAH and the Environment: Lessons Learned from Major Accidents 1999–2000*. Process Safety and Environmental Protection. **80**(1):40-46.
5. Hansell, A. and L. Beale, *Small Area Health Statistics Unit Study into health risks in the area exposed to the plume from the October 2000 chemical fire at CSG in Sandhurst, Gloucestershire*. 2011.

Supplementary Methods

Methods S1. Changes in Census Output Areas (COAs) between the 2001 and 2011 Censuses

The changes when moving from 2001 to 2011 COAs in the study area were that one COA (23UEGG0018) was split into two (E00168939 and E00168940) and two (23UGGP0014 and 23UGGP0015) were merged into a single COA (E00169004). Consequently, the extent of the study area remained constant and the number of COAs in the study area remained at nineteen.

Maps covering periods prior to 2011 use the COAs from the 2001 Census (beige background), whereas those subsequent to 2011 use the COAs from the 2011 Census (light blue background) (**Supplementary Figure S2**).

Supplementary Figures

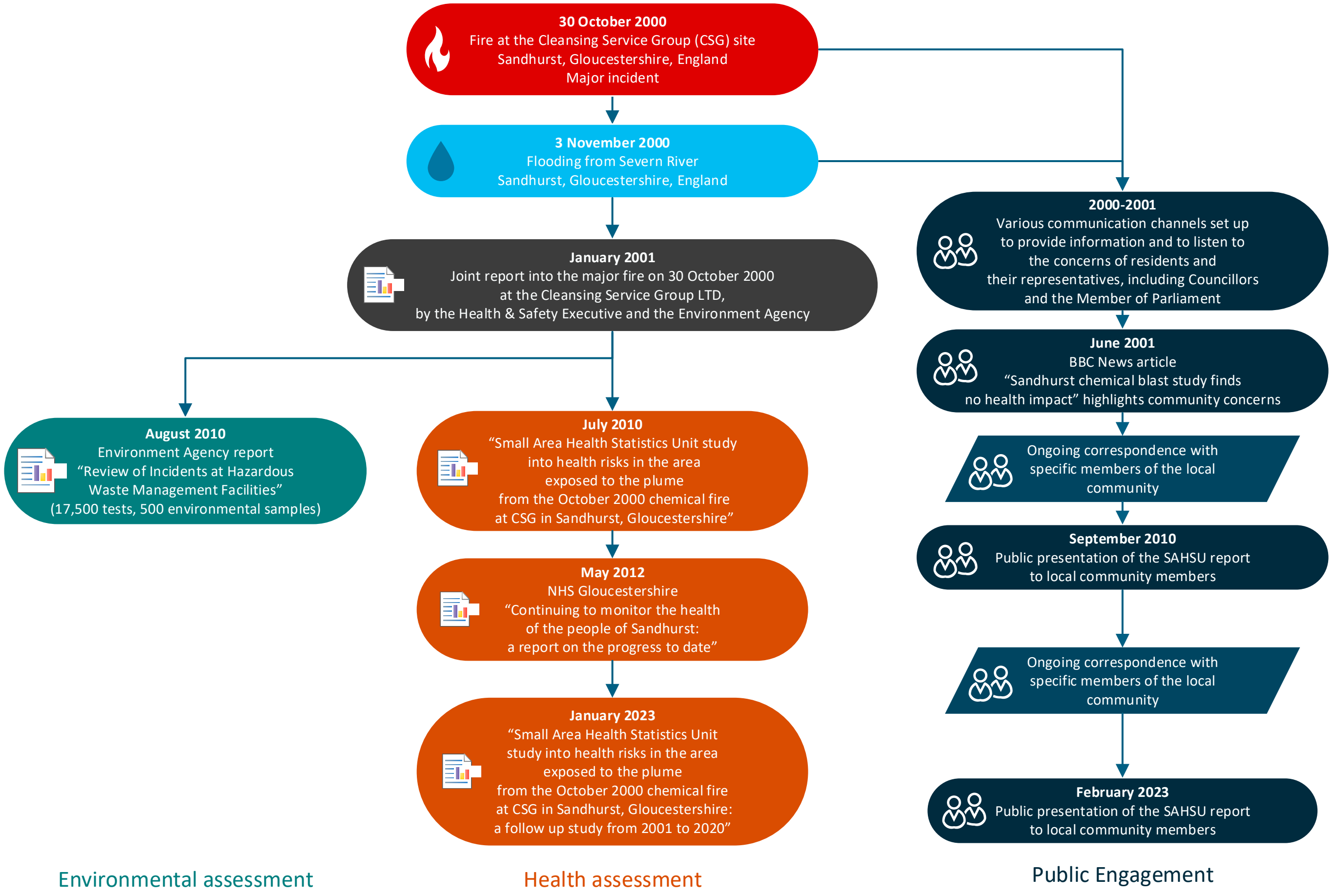

Figure S1. Timeline of the incident and main reports and public engagement activities relating to the large fire at the CGS facility in 2000 in Sandhurst, Gloucestershire, England.

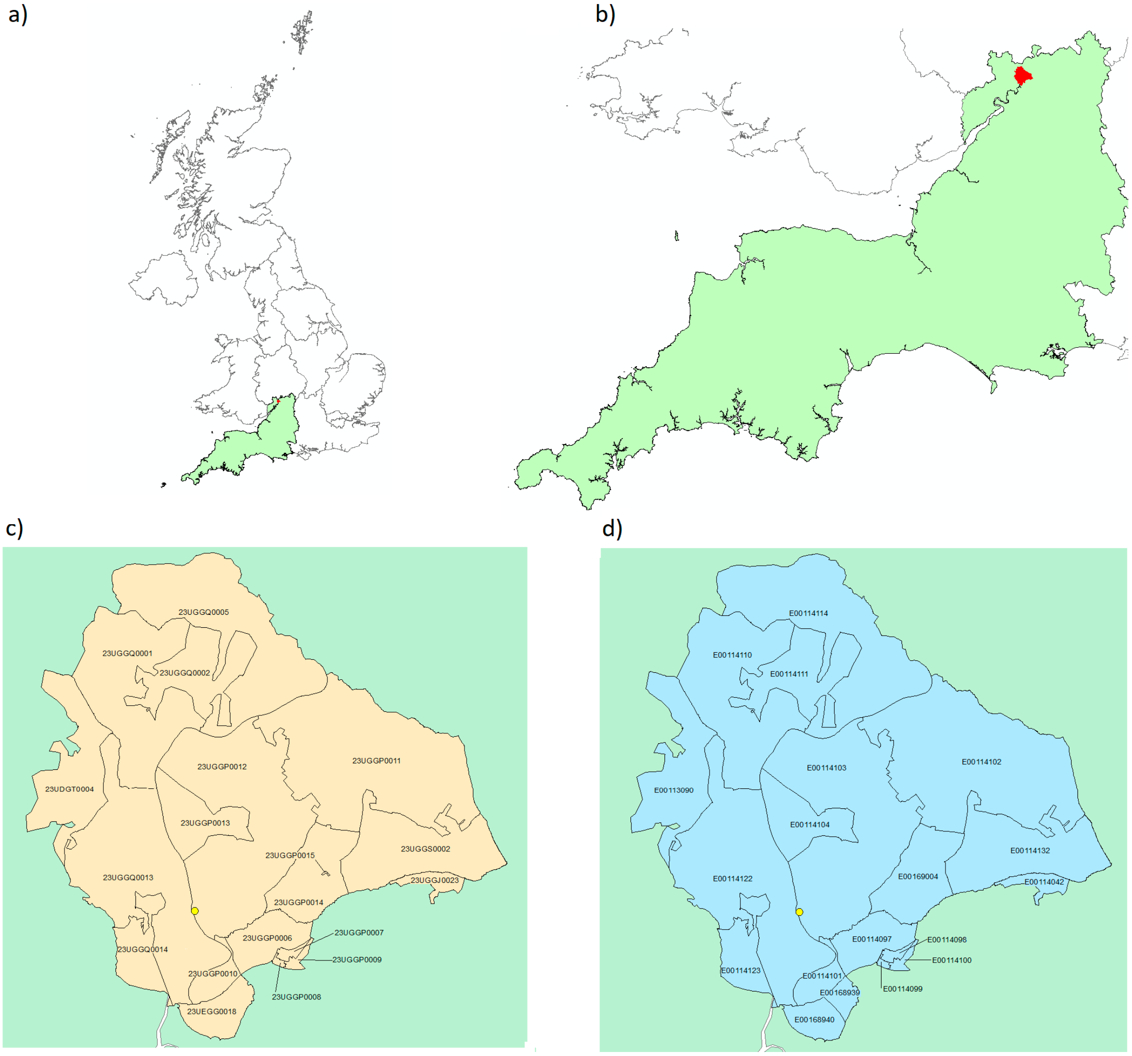

Figure S2. Maps of the study and comparison areas. a) Great Britain and Northern Ireland with the South-West (SW) region of England in green. b) SW region of England with the study area highlighted in red. c) Study area showing the 19 Census Output Areas (COAs) in 2001 with labels (used for analysis period prior to 2011). d) Study area showing the 19 COAs in 2011 with labels (used for analysis periods from 2011 onwards). Yellow dot indicates the site of the fire.

Supplementary tables

Table S1. Toxicity of the chemical substances involved in the fire at the CGS storage site, based the inventory included in the 2001 report of the Environment Agency and Health & Safety Executive [1]. The extent and nature of the health effect will depend on many factors including level of exposure and length of time exposed.

	Substance	Quantity (tonnes)	Toxicity	References
**1**	Cyanide solutions	0.1	Long-term exposure to low levels may lead to non-specific neurological symptoms, effects on the thyroid, and optic neuropathy. Hydrogen cyanide has no mutagenic properties and is not considered to be a carcinogen.	1
**2**	Pesticide residues	0.8	Long-term exposure to pesticides can lead to a range of health issues, including cancer, reproductive system damage, liver damage, and brain damage. The respiratory pathway occurs through breathing or inhalation and is caused by the pesticides' volatile components	2
**3**	Mercury based pesticide	0.2	Acute inhalation of elemental mercury may also cause GI effects such as stomatitis, abdominal pain, vomiting, diarrhoea and ulceration of the oral mucosa, as well as cardiovascular effects such as hypertension and tachycardia.	3
**4**	Triozine biocide	0.12	No chronic health effects known. Carcinogenicity not classifiable for human or animal.	4
**5**	Metals (Nickel, Copper, Aluminium)	0.8	Nickel: Chronic inhalation of nickel or nickel compounds can cause rhinitis, sinusitis, anosmia, and in extreme cases perforation of the nasal septum. The International Agency for Research on Cancer (IARC) classified nickel compounds as carcinogenic to humans (Group 1).<br>Copper: Breathing fumes of copper can lead to metal fume fever, which is characterised by symptoms such as fever, headache and tiredness, as well as cough, sore throat, tightening of the chest. Sometimes a metallic taste in the mouth, nausea, vomiting and blurred vision can occur. There is very limited data available on whether copper can cause cancer in humans.	5a<br>5b

			Aluminium: Symptoms that indicate the presence of higher amounts of aluminium in the human body are nausea, mouth ulcers, skin ulcers, skin rashes, vomiting, diarrhoea and arthritic pain. Aluminium exposure is probably a risk factor for the onset of Alzheimer disease (AD) in humans.	5c
**6**	Flammable smalls	0.8	Too generic	/
**7**	Ammonium chloride	1.0	Acute inhalation may initially cause upper respiratory tract irritation. Chronic inhalation is associated with increased cough, phlegm, wheeze, and asthma.	7
**8**	Paint thinners	49.2	Vapours may cause headache, fatigue, dizziness and nausea. Vapours in high concentrations are anaesthetic. Symptoms following overexposure may include the following: Headache. Fatigue. Dizziness. Central nervous system depression. No evidence that they can cause cancer.	8
**9**	Chlorinated solvents	21.1	Central nervous system, reproductive, liver, and kidney toxicity, and carcinogenicity	9
**10**	Acrylic resin	4.8	Gas or vapour in high concentrations may irritate the respiratory system. No evidence of carcinogenicity in animal studies.	10
**11**	Paint stripper	8.0	Breathing in the vapours over short periods can cause sluggishness, irritability, light-headedness, nausea and headaches. Prolonged exposure may cause cancer in humans.	11
**12**	Mixed aerosols	Unknown	Too generic	/
**13**	Mixed solvents	20.5	Repeated exposure to solvents can have long-term effects on your health. These may include dermatitis and neurotoxic effects. Other possible effects on health vary according to which solvent you are exposed to.	13a, 13b
**14**	Acidic resin	0.8	Gas or vapour in high concentrations may irritate the respiratory system. Carcinogenicity not known.	14
**15**	Magnesium oxide/nitride	0.8	Breathing magnesium oxide can irritate the eyes and nose. No data on carcinogenicity.	15
**16**	Aluminium chloride	0.6	Long-term exposure can cause damage to lungs. In some animal tests, aluminium chloride has shown	16

			developmental and reproductive toxicity. Aluminium chloride has not been found to be carcinogenic in humans.	
**17**	Methylated spirit	2.2	Irritation of the respiratory system. Irritation of eyes, nose and throat. Coughing. Lacrimation of eyes. No information on carcinogenicity.	17
**18**	Methanol	6.0	Long-term inhalation exposure to methanol has resulted in headaches and eye irritation in workers. Methanol is considered not to be a mutagen or carcinogen in humans. Methanol is considered not to be a reproductive toxicant in humans.	18
**19**	Adhesives	34.0	VOCs have carcinogenic and mutagenic properties and can cause migraines, irritation to the eyes, nasal passages, mouth, and lungs, and respiratory problems.	19
**20**	Lab smalls	8.8	Too generic	/
**21**	Isopropanol	12.0	Prolonged inhalation can result in systemic features. Due to the lack of human data and the limited data in animals, it is not known whether isopropanol causes cancer therefore the International Agency for Research on Cancer (IARC) considered it as being not classifiable.	21
**22**	Acetone	4.8	Inhalation may cause respiratory irritation and chest tightness at high concentration. Systemic features may also develop. Exposure to acetone has not been linked to the development of cancer.	22
**23**	Batteries	Unknown	See heavy metals (nickel, lead, mercury).	/
	TOTAL	177.42		

Reference

1. Heath and Safety Executive, *Report for the Deputy Prime Minister, the Right Hon John Prescott MP, into the major fire on 30 October 2000 at Cleansing Service Group Ltd Sandhurst*. 2001.

Table S2. Comparison of the methodology used for the 2011 and 2023 Small Area Health Statistics Unit (SAHSU) studies. Differences are highlighted in bold.

	2011 SAHSU study	2023 SAHSU study
**Study period**	**5 years** (2001-2005) and preceding periods (1989-1994 & 1995-2000 for mortality; 1991-1995 & 1996-2000 for cancer)	**20 years** (2001-2005, 2006-2010, 2011-2015, 2016-2020) and preceding periods (1991-1995, 1996-2000)
**Study area**	Area exposed to the fire plume (19 COAs)	Area exposed to the fire plume (19 COAs)
**Reference area**	South-West England	South West of England (17,625 COAs)
**Exposure considered**	Airborne (plume)	Airborne (plume)
**Exposure not considered**	Contamination through soil or water	Contamination through soil or water
**Health outcomes**	All cancers;<br>All-cause mortality<br>Hospital admissions for respiratory disease	All cancers;<br>All-cause mortality<br>Hospital admissions for respiratory disease
**Adjustments**	Deprivation (Carstairs);	Deprivation (Carstairs); **smoking (CACI, 2011-2020)**

COA: Census output area.

Table S3. Comparison of the study area with the South West of England. 2011 descriptive statistics of the demographic and socio-economic characteristics of the population in the study and comparison areas, based on 2011 Census data.

	**Study Area** 19 COAs	**Comparison Area, SW Region** 17,625 COAs
**Age Distribution, N (%)**		
0-9	440 (8.39%)	570,895 (10.8%)
10-19	567 (10.8%)	621,215 (11.7%)
20-29	551 (10.5%)	643,235 (12.1%)
30-39	543 (10.4%)	617,440 (11.7%)
40-49	789 (15.0%)	764,947 (14.4%)
50-59	811 (15.5%)	674,662 (12.7%)
60-69	752 (14.3%)	663,434 (12.5%)
70-79	522 (9.95%)	430,871 (8.14%)
80+	271 (5.17%)	308,886 (5.83%)
**Gender, N (%)**		
Female	2,658 (50.7%)	2,698,929 (51.0%)
Male	2,588 (49.3%)	2,596,656 (49.0%)
**Deprivation quintiles at COA level, N (%)[1]**		
Q1 – Least deprived	1,543 (29.4%)	962,859 (18.2%)
Q2	3,116 (59.4%)	1,280,378 (24.2%)
Q3	0 (0%)	1,366,409 (25.8%)
Q4	0 (0%)	1,213,490 (22.9%)
Q5 – Most deprived	587 (11.2%)	472,449 (8.92%)
**Smoking proxy (tobacco expenditure) at COA level[2]**		
Population aged 16 or over, N	4,480	4,353,916
Mean tobacco expenditure per week per person aged 16 or over	£4.86	£5.74

[1] Carstairs index socio-economic status from 2011 Census data, available at Census Output Area (COA) level

[2] CACI data on tobacco expenditure for 2011 (© Copyright 1996-2014 CACI Limited) available at COA level

Table S4. All cancers 1991-1995. Indirectly standardised relative risk for all cancer registrations in the study area 1991-1995. Excluding 85+ age categories.

Adjusted for Deprivation	Sex	Observed	Expected	Relative Risk	Lower 95% CI	Upper 95% CI
No	Male	55	60.65	0.91	070	1.18
	Female	52	65.91	0.79	0.60	1.04
	Both	107	126.55	0.85	0.70	1.02
Yes	Male	55	57.53	0.96	0.73	1.25
	Female	52	63.44	0.82	0.62	1.08
	Both	107	120.97	0.88	0.73	1.07

Table S5. All cancers 1996-2000. Indirectly standardised relative risk for all cancer registrations in the study area 1996-2000. Excluding 85+ age categories.

Adjusted for Deprivation	Sex	Observed	Expected	Relative Risk	Lower 95% CI	Upper 95% CI
No	Male	84	78.36	1.07	0.87	1.33
	Female	79	80.33	0.98	0.79	1.23
	Both	163	158.69	1.03	0.88	1.20
Yes	Male	84	76.63	1.10	0.89	1.36
	Female	79	78.91	1.00	0.80	1.25
	Both	163	155.54	1.05	0.90	1.22

Table S6. All cancers 2001-2005. Indirectly standardised relative risk for all cancer registrations in the study area 2001-2005. Excluding 85+ age categories.

Adjusted for Deprivation	Sex	Observed	Expected	Relative Risk	Lower 95% CI	Upper 95% CI
No	Male	84	106.01	0.79	0.64	0.98
	Female	92	99.88	0.92	0.75	1.13
	Both	176	205.90	0.85	0.74	0.99
Yes	Male	84	106.45	0.79	0.64	0.98
	Female	92	99.51	0.92	0.75	1.13
	Both	176	205.96	0.85	0.74	0.99

Table S7. All cancers 2006-2010. Indirectly standardised relative risk for all cancer incidence in the study area 2006-2010. Excluding 85+ age category.

Adjusted for Deprivation	Sex	Observed	Expected	Relative Risk	Lower 95% CI	Upper 95% CI
No	Male	145	132.04	1.10	0.93	1.29
	Female	115	116.58	0.99	0.82	1.18
	Both	260	248.62	1.05	0.93	1.18
Yes	Male	145	132.89	1.09	0.93	1.28
	Female	115	117.43	0.98	0.82	1.18
	Both	260	250.32	1.04	0.92	1.17

Table S8. All cancers 2011-2015. Indirectly standardised relative risk for all cancer incidence in the study area 2011-2015. Excluding 85+ age category.

Adjusted for Deprivation	Adjusted for Smoking Proxy	Sex	Observed	Expected	Relative Risk	Lower 95% CI	Upper 95% CI
No	No	Male	142	155.72	0.91	0.77	1.08
		Female	140	141.84	0.99	0.84	1.17
		Both	282	297.55	0.95	0.84	1.07
Yes	No	Male	142	155.85	0.91	0.77	1.07
		Female	140	141.96	0.99	0.84	1.16
		Both	282	297.81	0.95	0.84	1.06
Yes	Yes	Male	142	155.38	0.91	0.78	1.08
		Female	140	142.24	0.98	0.83	1.16
		Both	282	297.62	0.95	0.84	1.07

Table S9. All cancers 2013-2017. Indirectly standardised relative risk for all cancer incidence in the study area 2013-2017. Excluding 85+ age category.

Adjusted for Deprivation	Adjusted for Smoking Proxy	Sex	Observed	Expected	Relative Risk	Lower 95% CI	Upper 95% CI
No	No	Male	156	164.91	0.95	0.81	1.11
		Female	134	152.44	0.88	0.74	1.04
		Both	290	317.35	0.91	0.81	1.03
Yes	No	Male	156	165.02	0.95	0.81	1.11
		Female	134	152.49	0.88	0.74	1.04
		Both	290	317.51	0.91	0.81	1.03
Yes	Yes	Male	156	165.22	0.94	0.81	1.11
		Female	134	152.83	0.88	0.74	1.04
		Both	290	318.06	0.91	0.81	1.02

Table S10. All-cause mortality 1991-1995. Indirectly standardised relative risk (RR) of all-cause mortality, 1991-1995.

Adjusted for Deprivation	Sex	Observed	Expected	Relative Risk	Lower 95% CI	Upper 95% CI
No	Male	132	113.32	1.16	0.98	1.38
	Female	123	128.48	0.96	0.80	1.14
	Both	255	241.80	1.05	0.93	1.19
Yes	Male	132	104.27	1.27	1.07	1.50
	Female	123	124.74	0.99	0.83	1.18
	Both	255	229.01	1.11	0.98	1.26

Table S11. All-cause mortality 1996-2000. Indirectly standardised relative risk of all-cause mortality, 1996-2000.

Adjusted for Deprivation	Sex	Observed	Expected	Relative Risk	Lower 95% CI	Upper 95% CI
No	Male	130	120.33	1.08	0.91	1.28
	Female	121	122.87	0.99	0.82	1.18
	Both	251	243.20	1.03	0.91	1.17
Yes	Male	130	110.79	1.17	0.99	1.39
	Female	121	120.00	1.00	0.84	1.20
	Both	251	231.26	1.09	0.96	1.23

Table S12. All-cause mortality 2001-2005. Indirectly standardised relative risk for all-cause mortality, 2001-2005.

Adjusted for Deprivation	Sex	Observed	Expected	Relative Risk	Lower 95% CI	Upper 95% CI
No	Male	116	124.40	0.93	0.78	1.12
	Female	138	118.35	1.17	0.99	1.38
	Both	254	242.74	1.05	0.93	1.18
Yes	Male	116	114.40	1.01	0.85	1.27
	Female	138	114.75	1.20	1.02	1.42
	Both	254	229.15	1.11	0.98	1.25

Table S13. All-cause mortality 2006-2010. Indirectly standardised relative risk for all-cause mortality, 2006-2010.

Adjusted for Deprivation	Sex	Observed	Expected	Relative Risk	Lower 95% CI	Upper 95% CI
No	Male	118	123.47	0.96	0.80	1.15
	Female	115	112.21	1.03	0.85	1.23
	Both	233	235.68	0.99	0.87	1.12
Yes	Male	118	113.50	1.04	0.87	1.25
	Female	115	108.74	1.06	0.88	1.27
	Both	233	222.24	1.05	0.92	1.19

Table S14. All-cause mortality 2011-2015. Indirectly standardised relative risk for all-cause mortality, 2011-2015.

Adjusted for Deprivation	Adjusted for Smoking Proxy	Sex	Observed	Expected	Relative Risk	Lower 95% CI	Upper 95% CI
No	No	Male	168	138.28	1.22	1.04	1.41
		Female	154	132.68	1.16	0.99	1.36
		Both	322	270.96	1.19	1.07	1.33
Yes	No	Male	168	129.54	1.30	1.12	1.51
		Female	154	128.79	1.20	1.02	1.40
		Both	322	258.32	1.25	1.12	1.39
Yes	Yes	Male	168	126.16	1.33	1.15	1.55
		Female	154	127.30	1.21	1.03	1.42
		Both	322	253.47	1.27	1.14	1.42

Table S15. All-cause mortality 2016-2020. Indirectly standardised relative risk for all-cause mortality, 2016-2020.

Adjusted for Deprivation	Adjusted for Smoking Proxy	Sex	Observed	Expected	Relative Risk	Lower 95% CI	Upper 95% CI
No	No	Male	160	162.68	0.98	0.84	1.15
		Female	138	157.29	0.88	0.75	1.04
		Both	298	319.98	0.93	0.83	1.04
Yes	No	Male	160	152.37	1.05	0.90	1.23
		Female	138	151.49	0.91	0.77	1.08
		Both	298	303.86	0.98	0.88	1.10
Yes	Yes	Male	160	148.78	1.08	0.92	1.26
		Female	138	150.51	0.92	0.78	1.08
		Both	298	299.29	1.00	0.89	1.12

Table S16. Respiratory hospital episodes 1991-1995. Indirectly standardised relative risk of hospital episodes of respiratory disease, 1991-1995. Excluding 85+ age categories.

Adjusted for Deprivation	Sex	Observed	Expected	Relative Risk	Lower 95% CI	Upper 95% CI
No	Male	134	130.74	1.02	0.87	1.21
	Female	122	108.61	1.12	0.94	1.34
	Both	256	239.35	1.07	0.95	1.21
Yes	Male	134	114.32	1.17	0.99	1.39
	Female	122	95.95	1.27	1.06	1.52
	Both	256	210.27	1.22	1.08	1.38

Table S17. Respiratory hospital episodes 1996-2000. Indirectly standardised relative risk of hospital episodes of respiratory disease, 1996-2000. Excluding 85+ age categories.

Adjusted for Deprivation	Sex	Observed	Expected	Relative Risk	Lower 95% CI	Upper 95% CI
No	Male	117	150.62	0.78	0.65	0.93
	Female	133	128.71	1.03	0.87	1.23
	Both	250	279.33	0.90	0.79	1.01
Yes	Male	117	131.86	0.89	0.74	1.06
	Female	133	111.80	1.19	1.00	1.41
	Both	250	243.66	1.03	0.91	1.16

Table S18. Respiratory hospital episodes 2001-2005. Indirectly standardised relative risk of hospital episodes of respiratory disease, 2001-2005. Excluding 85+ age categories.

Adjusted for Deprivation	Sex	Observed	Expected	Relative Risk	Lower 95% CI	Upper 95% CI
No	Male	207	186.45	1.11	0.97	1.27
	Female	176	151.71	1.16	1.00	1.35
	Both	383	338.16	1.13	1.03	1.25
Yes	Male	207	160.84	1.29	1.12	1.48
	Female	176	130.51	1.35	1.16	1.56
	Both	383	291.35	1.32	1.19	1.45

Table S19. Respiratory hospital episodes 2006-2010. Indirectly standardised relative risk of hospital episodes of respiratory disease, 2006-2010. Excluding 85+ age categories.

Adjusted for Deprivation	Sex	Observed	Expected	Relative Risk	Lower 95% CI	Upper 95% CI
No	Male	259	231.16	1.12	0.99	1.27
	Female	201	186.13	1.08	0.94	1.24
	Both	460	417.29	1.10	1.01	1.21
Yes	Male	259	198.85	1.30	1.15	1.47
	Female	201	160.10	1.26	1.09	1.44
	Both	460	358.96	1.28	1.17	1.40

Table S20. Respiratory hospital episodes 2011-2015. Indirectly standardised relative risk of hospital episodes of respiratory disease, 2011-2015. Excluding 85+ age categories.

Adjusted for Deprivation	Adjusted for Smoking Proxy	Sex	Observed	Expected	Relative Risk	Lower 95% CI	Upper 95% CI
No	No	Male	357	297.22	1.20	1.08	1.33
		Female	356	246.76	1.44	1.30	1.60
		Both	713	543.98	1.31	1.22	1.41
Yes	No	Male	357	273.00	1.31	1.18	1.45
		Female	356	231.74	1.54	1.39	1.70
		Both	713	504.74	1.41	1.31	1.52
Yes	Yes	Male	357	263.57	1.35	1.22	1.50
		Female	356	222.49	1.60	1.44	1.78
		Both	713	486.06	1.47	1.36	1.58

Table S21. Respiratory hospital episodes 2016-2020. Indirectly standardised relative risk of hospital episodes of respiratory disease, 2016-2020. Excluding 85+ age categories.

Adjusted for Deprivation	Adjusted for Smoking Proxy	Sex	Observed	Expected	Relative Risk	Lower 95% CI	Upper 95% CI
No	No	Male	443	397.81	1.11	1.02	1.22
		Female	465	354.48	1.32	1.20	1.44
		Both	908	752.29	1.21	1.13	1.29
Yes	No	Male	443	366.00	1.21	1.10	1.33
		Female	465	329.53	1.41	1.29	1.55
		Both	908	695.53	1.31	1.22	1.39
Yes	Yes	Male	443	353.98	1.25	1.14	1.37
		Female	465	319.33	1.46	1.33	1.60
		Both	908	673.31	1.35	1.26	1.44
